# Reproducing Infra-Slow Oscillations with Dopaminergic Modulation

**DOI:** 10.1038/s41598-017-02366-z

**Published:** 2017-05-25

**Authors:** Toshihiro Kobayashi, Yutaka Shimada, Kantaro Fujiwara, Tohru Ikeguchi

**Affiliations:** 1 Department of Management Science, Graduate School of Engineering, Tokyo University of Science, 6–3–1 Niijuku, Katsushika-ku, Tokyo Japan; 2 Department of Information and Computer Technology, Faculty of Engineering, Tokyo University of Science, 6–3–1 Niijuku, Katsushika-ku, Tokyo Japan

## Abstract

In the human brain, billions of neurons construct a neural network via synaptic connections. Neuronal excitation and inhibition are transmitted to other neurons through synapses via neurotransmitters. Dopamine is one of these neurotransmitters that plays a number of important roles. There are a variety of rhythms in the brain, such as alpha rhythm, beta rhythm, and so on. Infra-slow oscillation, ISO, is one of the rhythms observed in the brain, and ranges below 0.1 Hz. One of the key roles of dopamine is the generation of ISO in neural networks. Although the mechanism underlying the generation of ISO remains unknown, ISO can be generated by activation of the D1-type dopamine receptor. The D1-type receptor regulates spike timing-dependent plasticity (STDP), which is a learning rule of the change in synaptic weights. In this paper, to reproduce ISO in neural networks, we show that dopaminergic modulation of STDP is essential. More specifically, we discovered a close relationship between two dopaminergic effects: modulation of the STDP function and generation of ISO. We therefore, numerically investigated the relationship in detail and proposed a possible mechanism by which ISO is generated.

## Introduction

Billions of neurons are present in the human brain and they exchange signals across synapses. The interaction between neurons generates a variety of rhythms, such as alpha (8–12 Hz), beta (12–30 Hz), and gamma (30–70 Hz) rhythms. Among them, infra-slow oscillation (ISO, <0.1 Hz) is an important rhythm of brain activity. ISO was discovered by Aladjalova with a local field potential recorded from the rabbit neocortex^1^. Since then, ISO has been observed in the brains of various kinds of mammals^2–5^. However, the mechanism underlying the generation of ISO is not understood in detail.

Although the detailed mechanism is unknown, several conditions involved in the generation of ISO have been reported. Ruskin *et al*. have shown that the dopamine agonist apomorphine induces a firing rate that oscillates with a large amplitude and remarkably slow frequency (<0.1 Hz)^6^. They found infra-slow oscillations in the firing rate with substantia nigra dopaminergic neurons in the basal ganglia of rats induced by these D1 and D2-type receptor agonists.

What type of dopaminergic effects can induce ISO? Recently, it has been found that some neurotransmitters affect synaptic plasticity. Generally, the change in the synaptic weights depends on spike timing called spike-timing-dependent plasticity (STDP)^7^. When a presynaptic spike causes a postsynaptic neuron to fire, the synapse is potentiated (this is called long term potentiation, LTP). When the presynaptic spikes do not cause the postsynaptic neurons to fire, the synapse is depressed (this is called long term depression, LTD). The synaptic weight changes markedly when the presynaptic and postsynaptic neurons fire with a small difference in spike timing. The STDP window changes its shape due to the effects of neurotransmitters, such as dopamine, for instance, Yang and Dani showed that activation and inhibition of dopamine receptors change the shape of the STDP window^8^. They investigated how the activation of D1 and D5-type dopamine receptors regulates the STDP of the medial perforant path (mPP) synapse onto dentate granule cells. Compared to the STDP window without dopaminergic effect, when D1-type receptors are activated, larger LTP and LTD are induced with a small timing difference, and smaller LTP and LTD are induced with a larger spike-timing difference.

To investigate the relationship between the variability of the STDP window function and the generation of ISO, we conducted computational experiments. More specifically, we constructed a neural network model with an STDP rule affected by dopaminergic modulation. To model the effect of D1 receptor activation, we changed the width of the STDP window in the temporal direction. By narrowing the STDP window in the temporal direction, we show that a neural network model with STDP can reproduce ISO.

## Results

### Temporal Change in Firing Rates

We used the Izhikevich neuron model^9^ as a processing element of a neural network model. The network consists of 800 excitatory neurons and 200 inhibitory neurons, and each connection has conduction delay under 20 [ms]. The details of the neural network model are described in the Methods. We used the STDP rule for learning of the neural network. In STDP, the magnitude of the rate of the change in synaptic weights depends on the timing of spikes: if a presynaptic spike arrives at the postsynaptic neuron before the postsynaptic neuron fires, the synapse is potentiated (LTP). If the presynaptic spike arrives at the postsynaptic neuron after the postsynaptic neuron fired, the synapse is depressed (LTD). The magnitude of change in synaptic weights is decided by the STDP function, which is, in turn, defined by Eq. (1)^7^,

1 $${\rm{\Delta }}{w}_{ij}({\rm{\Delta }}{t}_{ij})=\{\begin{array}{c}{A}_{+}\exp (-\frac{{\rm{\Delta }}{t}_{ij}}{\tau })({\rm{\Delta }}{t}_{ij} > 0),\\ -{A}_{-}\exp (\frac{{\rm{\Delta }}{t}_{ij}}{\tau })({\rm{\Delta }}{t}_{ij} < 0),\end{array}$$

where Δ*t*_*ij*_ = *t*_*i*_ − *t*_*j*_ − *δ*_*ij*_, *t*_*i*_ is the firing time of the postsynaptic neuron *i*, *t*_*i*_ is the firing time of the presynaptic neuron *j*, *δ*_*ij*_ is the conduction delay from neuron *j* to neuron *i*, *A*_+_ is the maximum value of LTP, *A*_−_ is the maximum value of LTD, and *τ* is the time constant of LTP and LTD. To reproduce dopaminergic modulation, we changed the width of the STDP window by changing the value of *τ*. Although synapses are heterogeneous *in vivo*, we used a homogeneous STDP for all synapses, for the sake of simplicity.

**Figure 1:**
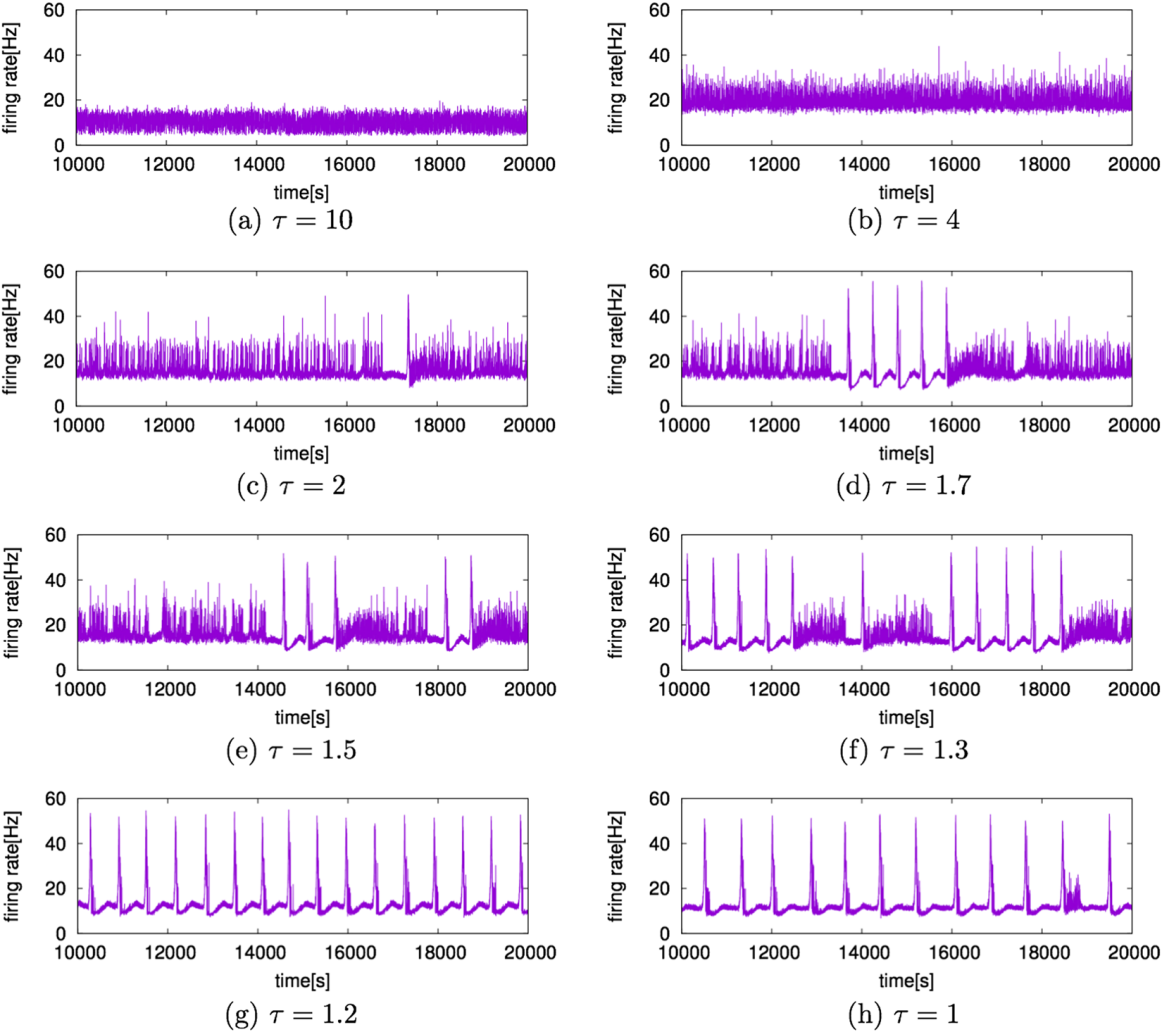
Temporal changes in firing rates. The horizontal axis is time [s] and the vertical axis is the firing rate [Hz]. When *τ* = 10, the firing rate oscillates with high frequency with an almost constant amplitude. When the values of *τ* become smaller, as in (**b**) and (**c**), the amplitudes of the firing rate become larger. When *τ* ≤ 1.7, as in (**d**), (**e**), and (**f**), the amplitudes change irregularly, exhibiting sudden increases in the firing rates. When *τ* takes a value close to unity, as shown in (**g**) and (**h**), the firing rate periodically repeats sudden increases and decreases with very slow frequency (<0.002 Hz).

To apply dopaminergic modulation^8^ to STDP learning, we narrowed the STDP window by decreasing the value of *τ*. Figure 1 shows a time series of the mean firing rates when the value of *τ* is decreased from 10 to 1. We defined the firing rate as the average firing frequency of a single neuron among all neurons, for one second. More specifically, we defined the firing rate by *m*/*N* [Hz], where *m* firings are observed from *N* neurons per second. When *τ* = 10 (Fig. 1(a)), the firing rate fluctuated near 10 Hz with small amplitude. When the value of *τ* becomes smaller, the amplitude of the firing rate becomes larger (Fig. 1(b,c)). By decreasing the value of *τ* further, the amplitudes change irregularly and exhibit sudden increases in the firing rates (Fig. 1(d–f)). When *τ* ≤ 1.2, sudden increases in firing rates appeared periodically (Fig. 1(g,h)). Thus, by applying dopaminergic modulation^8^ to STDP learning, we can reproduce the slow oscillation of the firing rate caused by dopamine activation^6^.

**Figure 2:**
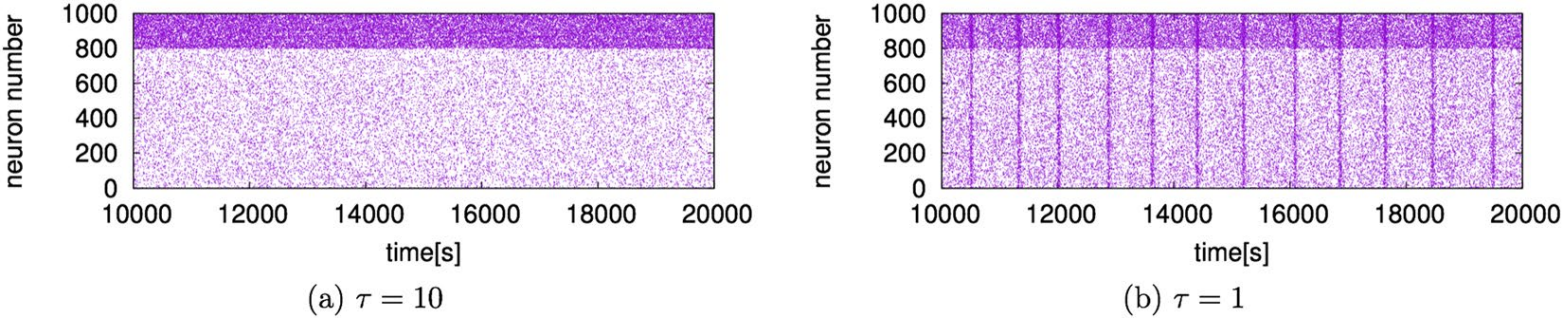
Raster plots when (**a**) *τ* = 10 [ms] and (**b**) *τ* = 1 [ms]. The horizontal axis is time [s]. The vertical axis is the neuron number: the numbers 1–800 correspond to excitatory neurons and the numbers 801–1000 correspond to inhibitory neurons.

Figure 2 shows the raster plots when the parameter *τ* takes the values *τ* = 10 [ms] and *τ* = 1 [ms]. As shown in Fig. 2, when *τ* = 10, no rhythmic activity of neurons is observed. However, when *τ* = 1, neurons fire synchronously with a very slow rhythm (at approximately 0.00125 Hz).

The maximum value of LTP, *A*_+_, is fixed to 0.1 and the maximum value of LTD, *A*_−_, is fixed to 0.12 in this paper. However, we can observe ISO, if we changed the values of *A*_+_ and *A*_−_ (See the Supplementary Information for the details).

### Temporal Change in Synaptic Weights

**Figure 3:**
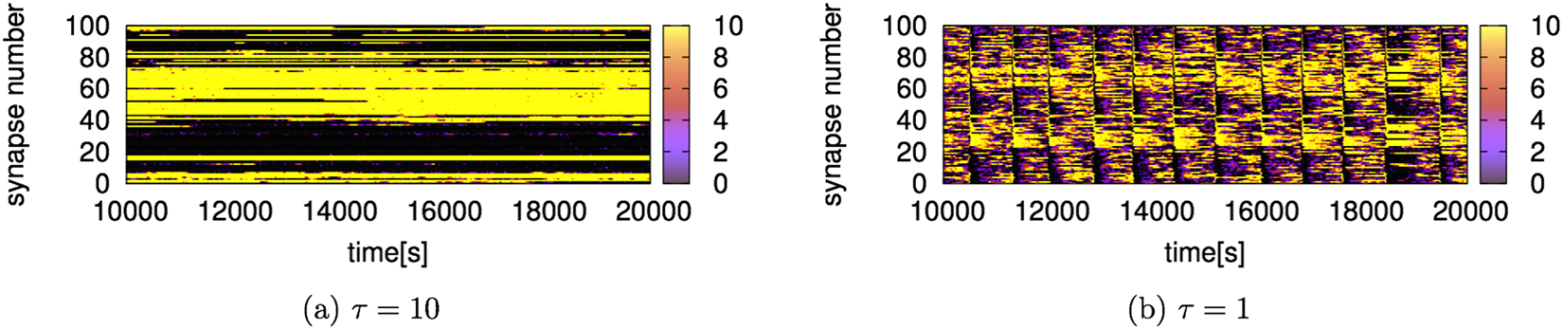
Temporal changes in the synaptic weights in all synapses from an excitatory neuron. The horizontal axis is time [s]. The vertical axis is the synapse index. The colors show the strength of synaptic weights indicated by color bars. The synaptic weights are almost constant and are separated into maximum and minimum values when *τ* = 10. On the other hand, the synaptic weights change periodically and some synapses have intermediate values when *τ* = 1.

To reveal the mechanism by which dopaminergic activation generates ISO, we investigated the temporal changes in the synaptic weights of each neuron when *τ* = 10 and *τ* = 1. Figure 3 shows the temporal change in all synaptic weights from an excitatory neuron arbitrarily selected. As shown in Fig. 3(a), when *τ* = 10, the synaptic weights are almost constant and are separated into a maximum value and a minimum value. However, when *τ* = 1 (Fig. 3(b)), the synaptic weights change with almost the same period as the firing rate. Some synapses have intermediate values as well as maximum and minimum values.

**Figure 4:**
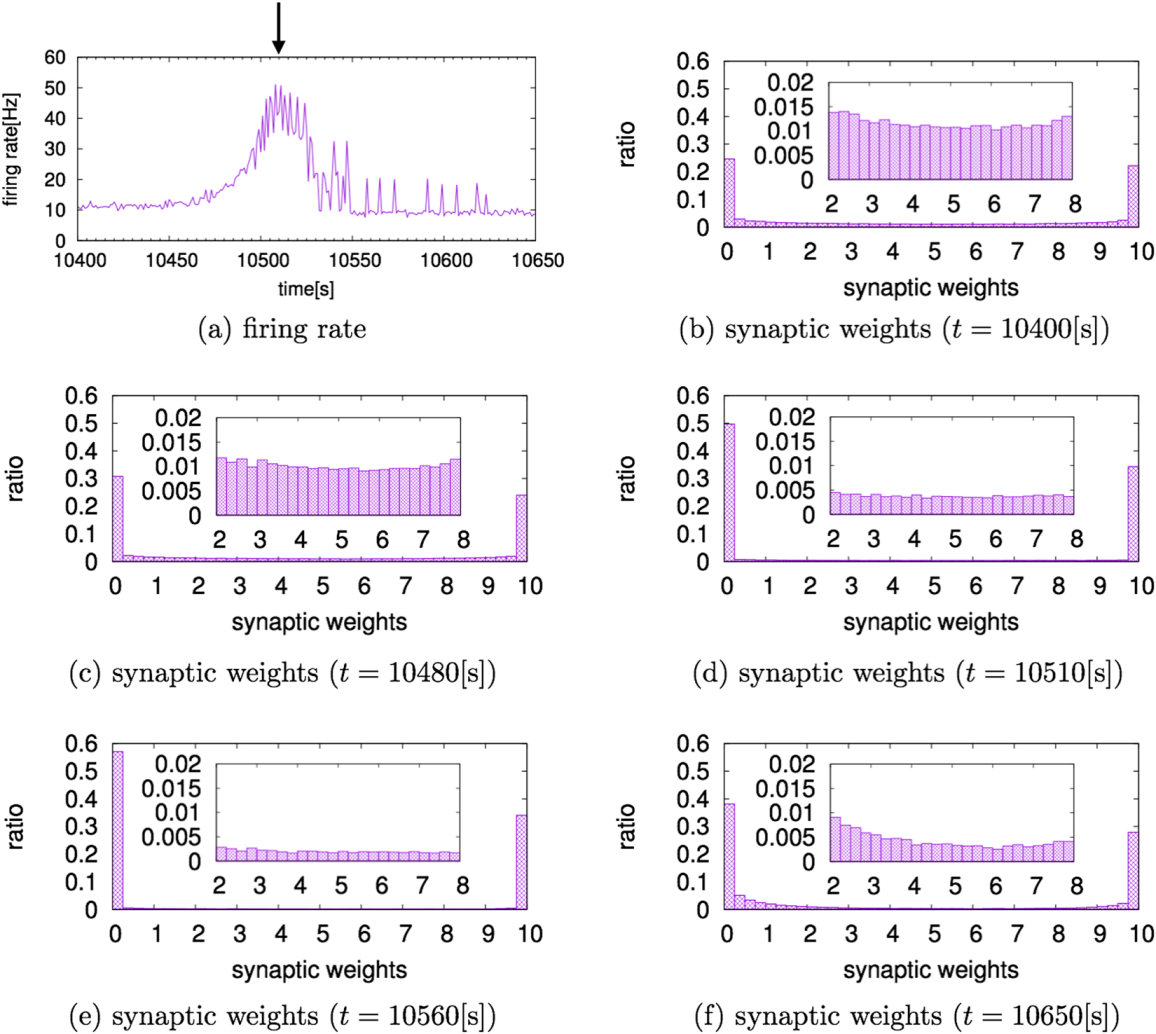
Changes in the firing rate and the frequency distribution of synaptic weights when *τ* = 1. (**a**) Time series of firing rates during a single sudden increase and decrease in the firing rate. The arrow shows a peak of the firing rates. (**b**–**f**) The frequency distribution of synaptic weights when the firing rate shows characteristic behaviors. The enlargements of each figure are shown to see the lower ratios of intermediate synaptic weights. When the firing rate is low and stable, as shown in (**b**), the distribution of synaptic weights is not fully separated into maximum and minimum weight values. When the firing rate begins to rise, as shown in (**c**), the number of synapses with the minimum weight value begins to increase. Then, the ratio of synapses taking maximum weight values also increases slightly. (**d**) The distribution of synaptic weights at the peak of the firing rate. After the peak, as shown in (**e**), the number of synapses taking maximum and minimum weight values continue to increase for a while. Once the firing rate again becomes low and stable, as shown in (**f**), the ratio of synapses with intermediate weight values again increases.

Figure 4 shows the change in the firing rate and the frequency distribution of synaptic weights when the ISO was observed with *τ* = 1. When *τ* = 1, that is, when the STDP window is narrow in the temporal direction, the neural network has less chance for learning than neural networks with a large value of *τ*. Therefore, when *τ* = 1, the distribution of synaptic weights is not completely separated into the maximum value and the minimum value, and intermediate synaptic weights are found. This tendency was observed when the firing rate was low and stable (*t* = 10,400 [s]), as shown in Fig. 4(b). However, the synaptic weights are not constant, and the synaptic weights of intermediate values change their values easily. Therefore, when the synaptic weight value is increased, the number of firing postsynaptic neurons increases, and the firing rate increases (*t* = 10,480 [s]). When the firing rate increases, the chance for learning increases and the number of synapses with the minimum weight value escalates (Fig. 4(c)). Then, the number of synapses taking the maximum weight value gradually increases. The number of the minimum value increases faster than that of the maximum value, because the amplitude of LTD is larger than that of LTP in the STDP function. Figure 4(d) shows the distribution of synaptic weights at the peak of the firing rate (*t* = 10,510 [s] in Fig. 4(a)). After the peak, the number of synapses with the maximum and minimum values continues to increase for a while (Fig. 4(e)). However, the firing rate begins to fall due to the increased number of synapses with minimum weights. The distribution that is separated into the maximum and minimum values collapses after the firing rate falls, because the weight values with the dichotomized distribution were generated by a large number of firings that are not relevant to the low firing rate. Therefore, once the firing rate again becomes low and stable, the number of synapses with an intermediate weight value again increases (Fig. 4(f)).

To investigate the change in the frequency distribution when the value of *τ* is changed, we used the Kullback-Leibler (KL) divergence^10^. The KL divergence is a measure of the difference between two probability distributions *p* and *q*. The value of KL divergence becomes large for distributions with different shapes and the value becomes small for distributions with similar shapes. When *p* = *q*, the value of KL divergence is 0. The KL divergence is expressed by Eq. (2):

2 $$D(p,q)=\sum _{i=1}^{M}({p}_{i}\,\mathrm{log}\,\frac{{p}_{i}}{{q}_{i}}),$$

**Figure 5:**
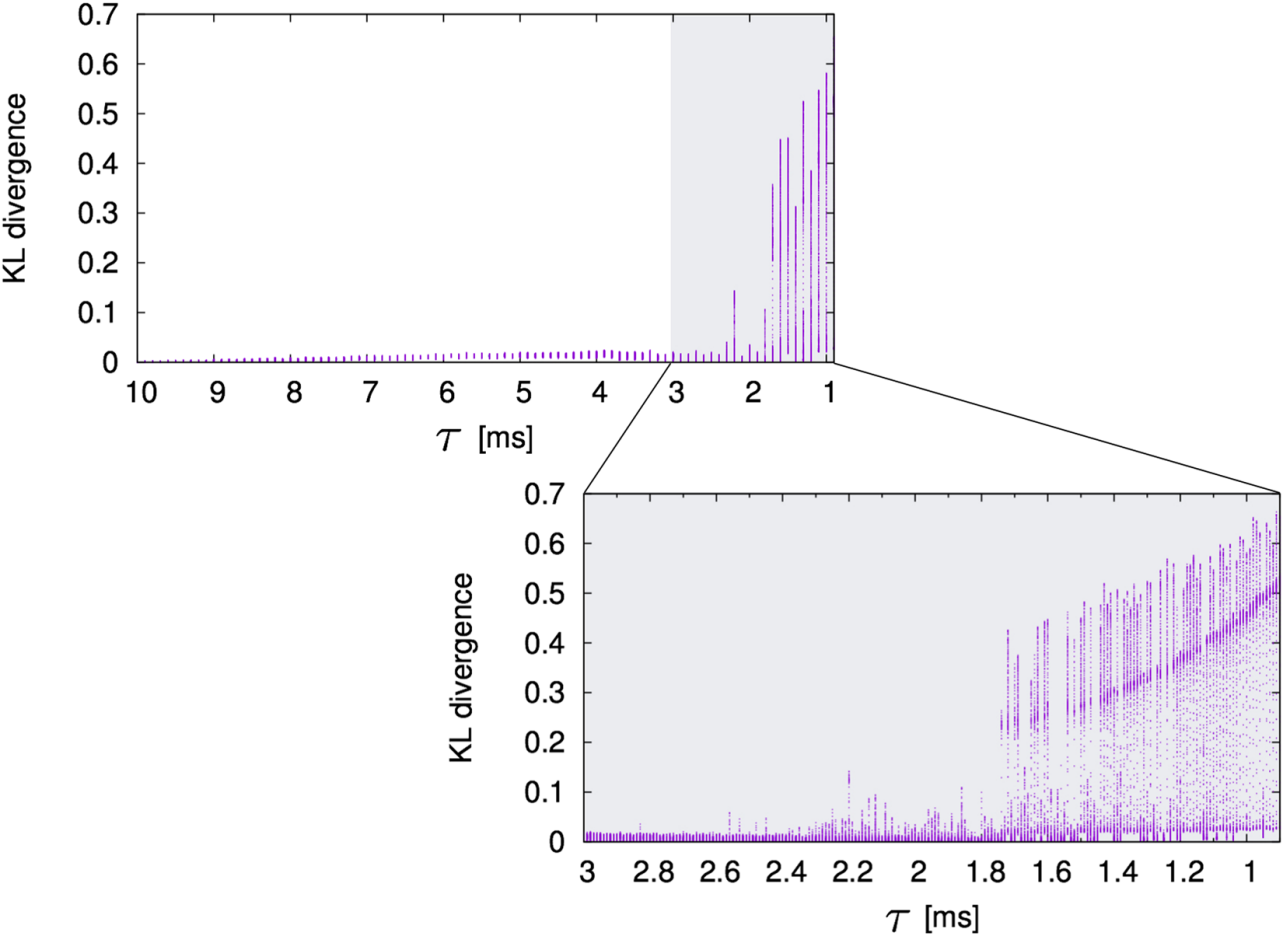
Change of the KL divergence between the probability distribution of the synaptic weights when *τ* = 10 [ms] with *t* = 1,000 [s] (reference distribution) and the probability distribution of each *τ* with each second. The horizontal axis is the value of parameter *τ*, and the vertical axis is the KL divergence. Values of the KL divergence are plotted for several temporal indexes simultaneously in the vertical direction.

In this paper, *p* is the probability distribution of synaptic weights when *τ* = 10 [ms] and *t* = 1,000 [s], *q* is the probability distribution of synaptic weights of each *τ* with each second. *M* is the number of classes for the probability distribution. Figure 5 represents the change in the frequency distribution measured by the KL divergence. Figure 5 shows the KL divergence when the value of *τ* was changed from *τ* = 10 to *τ* = 1. An enlargement of the figure when *τ* = 3 to *τ* = 1 is also shown in Fig. 5. We calculated the KL divergence between the probability distribution of synaptic weights when *τ* = 10 [ms] with *t* = 1,000 [s] (when learning is converged) and the probability distribution of synaptic weights of each *τ* with each time. We used the probability distribution of synaptic weights when *τ* = 10 [ms] and *t* = 1,000 [s] as a reference distribution, and calculated the distance between the reference distribution and the distribution of each *τ* with each second. We plotted the values of KL divergence of several temporal indexes simultaneously for each value of *τ* (Fig. 5). Then, if the values of the KL divergence take a particular value, it means that the probability distribution of synaptic weights hardly changes. As shown in Fig. 5, when the value of *τ* is decreased, the KL divergence takes almost the same value from *τ* = 10 to *τ* = 3. On the other hand, when the values of the KL divergence take various values, it means that the probability distribution of synaptic weights changes largely. As shown in Fig. 5, from *τ* = 1.7 to *τ* = 1, the values of the KL divergence change markedly. Namely, as the value of *τ* becomes smaller, the change in probability distribution of synaptic weights becomes larger. In particular, when ISO is reproduced in the neural network, we can observe large changes in the probability distribution of synaptic weights.

### Histogram of Spike-Timing Difference

**Figure 6:**
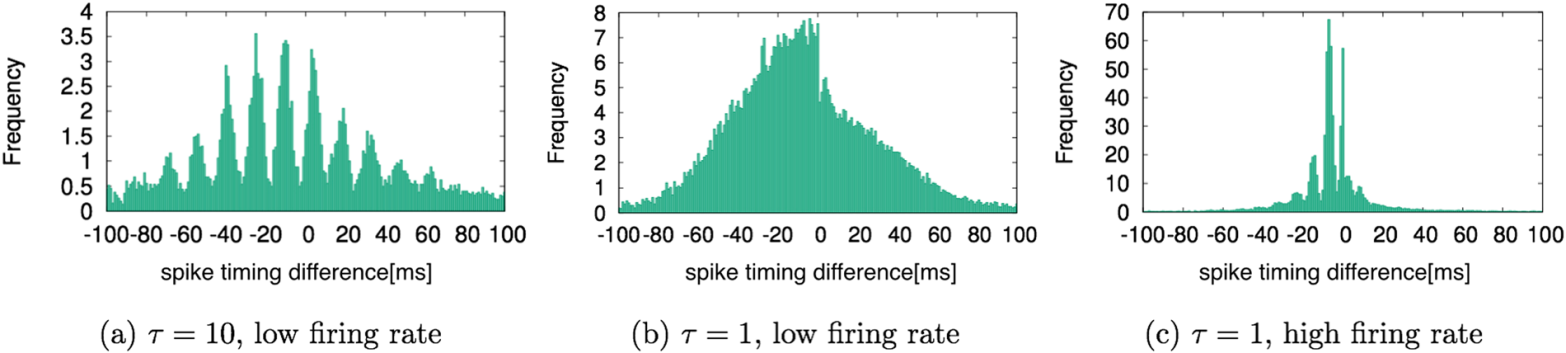
Frequency distributions of spike-timing differences between presynaptic and postsynaptic neurons when (**a**) *τ* = 10, (**b**) *τ* = 1 during a low firing rate and (**c**) *τ* = 1 during a high firing rate. The horizontal axis represents difference in spike-timing. The vertical axis represents frequency.

To analyze the relationship between the firing of presynaptic neurons and the firing of postsynaptic neurons, we investigated the spike-timing differences between presynaptic and postsynaptic neurons. Figure 6(a) shows the frequency distribution of the spike-timing difference when *τ* = 10. As shown in Fig. 6(a), the frequency of the spike-timing difference repeats high and low values periodically. This rhythm is about 70 Hz, which corresponds to the gamma rhythm. The appearance of the gamma rhythm has already been shown in numerical experiments by Izhikevich^11^. This trend is independent of time. Therefore, the results shown in Fig. 6(a) are consistent with experiments by Izhikevich^11^.

Figure 6(b) and (c) shows the histograms of the spike-timing differences when *τ* = 1. Figure 6(b) illustrates the frequency distribution when the firing rate is low, and Fig. 6(c) represents the frequency distribution when the firing rate is high. As shown in these figures, more spike-timing differences occurred with negative values than with positive values. This means that LTD occurs more frequently than LTP. In particular, with high firing rates, the amount of LTD increases markedly (Fig. 6(c)).

LTD occurs more frequently than LTP due to the values of parameter *A*_+_ and *A*_−_ in Eq. (1). In this experiment, *A*_−_ is larger than *A*_+_ because there are several experimental evidences that LTD of STDP window is larger than LTP^12, 13^. Then, a large number of synapses are depressed and therefore LTD becomes to occur more frequently in the synapses. When the firing rate is high, such a tendency becomes remarkable.

### Change in the amount of LTP and LTD

**Figure 7:**
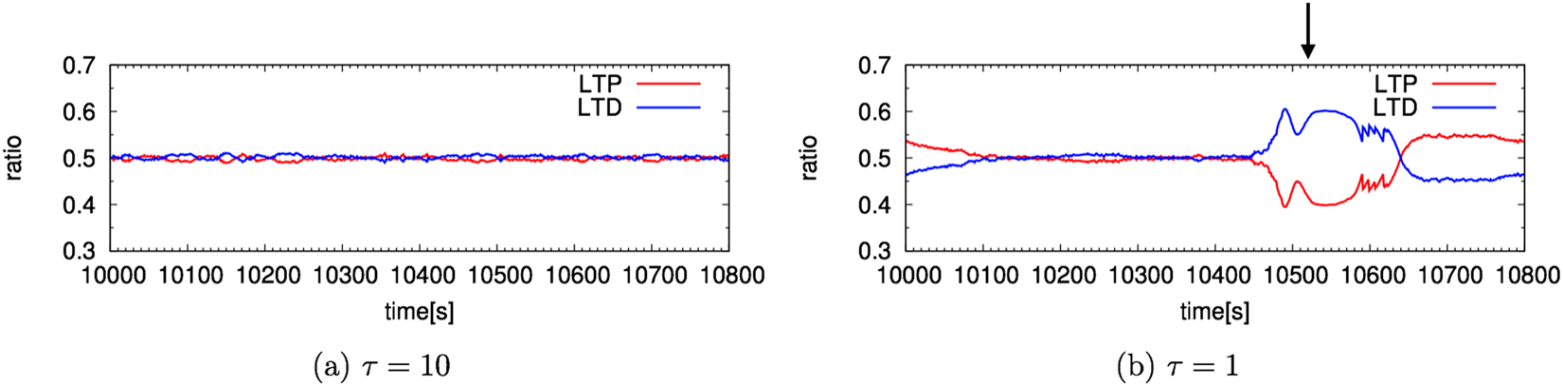
Temporal change in the ratio of amount of LTP and LTD. The change when (a) *τ* = 10 and (b) *τ* = 1. The arrow corresponds to the peak of firing rates shown in Fig. 4(a). The horizontal axis represents time [s]. The vertical axis represents the ratio of LTP and LTD.

Figure 7 shows the temporal change in the ratios of amount of LTP and LTD every second. As shown in Fig. 7, when *τ* = 10, the ratios of LTP and LTD take almost constant values. On the other hand, when *τ* = 1, the ratio of LTP (LTD) decreases (increases) near 10,500 [s], which corresponds to sudden increases in the firing rates, as shown in Fig. 4(a). From these results, we confirmed that when the sudden rise and fall of firing rates occur, the ratios of LTP and LTD also change largely.

### Change in Synaptic Weights that Induce Neuronal Firing

When *τ* = 1, a large amount of LTD occurs when the firing rate is high. We investigated why the firing rate is high despite the large number of LTD.

**Figure 8:**
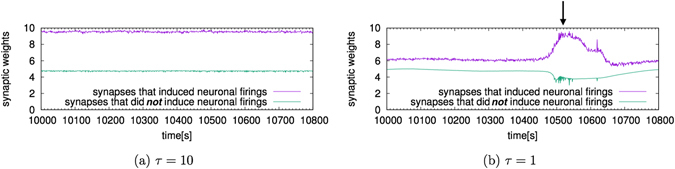
Temporal change in synaptic weights that induced neuronal firing. The horizontal axis represents time [s]. The vertical axis represents the synaptic weights. (**a**) The change when *τ* = 10 and (**b**) the change when *τ* = 1. The arrow shows a peak of the firing rates. When *τ* = 10, the two average synaptic weights fluctuate almost stationarily. When *τ* = 1, the synapses that induced firing become stronger and the synapses that did *not* induce firing become weaker near 10,500 [s]. This is simultaneous with the sudden rise of the firing rates (Fig. 4(a)) and the increase of the average amount of LTD among all synapses (Fig. 7(b)). Input from specific synapses can cause the firing rate to be high even if the amount of LTD is large.

Each neuron receives a number of inputs through synapses. However, such inputs do not always cause the neuron to fire. Then, we focused on the synapses that induced neuronal firing. We plotted a time series of the average synaptic weights in such synapses. Figure 8 shows the temporal change in the average value of the synaptic weights in the synapses that did induce neuronal firing and synapses that did *not* induce neuronal firing. As shown in Fig. 8(a), when *τ* = 10, the average synaptic weight fluctuates almost stationarily. When *τ* = 1, the synaptic weight that induced firing becomes stronger near 10,500 [s], which corresponds to sudden increases in the firing rates as shown in Fig. 4(a). On the other hand, the synaptic weight that did *not* induce firing becomes weaker at the same time. This result indicates that the firing rates can be high due to inputs from specific synapses, even if there was more LTD than LTP on average.

## Discussion

The oscillation of the firing rate shown in Fig. 1 is explained by the dynamics of synaptic weights. When the value of *τ* is large, that is, when the D1-type receptor is not activated^8^, the STDP window has a large width in the temporal direction. STDP learning with such a wide window affords many opportunities for learning and the synaptic weights are separated into maximum and minimum values. Because the bimodal distribution does not change its shape easily by LTP and LTD, the learning converges and the firing rate becomes stable. For instance, when *τ* = 10, the amounts of LTP and LTD are almost constant (Fig. 7(a)) and the firing rate is stable (Fig. 1(a)).

On the other hand, when the value of *τ* is small, that is, when the D1-type receptor is activated^8^, the STDP window is narrow in the temporal direction. STDP learning with such a narrow window affords fewer opportunities for learning and the synaptic weights are not fully separated into maximum and minimum values. Because the synaptic weights of intermediate values change their values easily by LTP and LTD, the learning does not converge. The firing rates are markedly influenced by the synaptic weights and exhibit sudden increases and decreases. For instance, when *τ* = 1, the amounts of LTP and LTD change their values largely (Fig. 7(b)) and the firing rates also change their values largely at the same time (Fig. 1(h)). Due to the sudden change in the firing rates, the balance in synaptic weights breaks down significantly. Subsequently, the values of the synaptic weights change with time by STDP learning, which again causes sudden changes in firing rates. These processes occur with a period of hundred seconds because the synaptic weights change their values very slowly due to the narrow STDP window. By repeating these processes, ISO is generated.

In this paper, we changed the value of *τ* because we assume that the essence of generation mechanism of ISO is the width of the learning window in temporal direction. Results show that we can explain simply the mechanism only by *τ*. However, it is important to examine in physiologically plausible condition close to the study by Yang & Dani^8^. To make the condition more physiologically plausible, we can change the learning window in vertical direction as well as temporal direction by changing the values of *A*_+_ and *A*_−_. The results show that we can reproduce ISO in more physiologically plausible condition (See the Supplementary Information for the details).

We reproduced ISO by constructing a neural network model with the STDP rule affected by dopaminergic modulation. By conducting the numerical experiments, we revealed that a narrowed STDP window induces the generation of ISO. The results obtained from our experiments indicated that ISO generated by D1-type receptor activation^6^ is a consequence of a number of synapses taking intermediate weight values, caused by an STDP window narrowed in the temporal direction. Our findings link seemingly disparate dopaminergic effects, highlighting an important mechanism by which dopamine affects motor and cognitive processes.

We do not have a clear answer for the biological relevance of our results of ISO, because the detailed phenomena related to ISO have not been revealed yet. This is partly due to the fact that too slow rhythms are often cutoff by filtering and then are not measured. However, it is also known that ISO is observed while sleeping or under the effect of the anesthesia^2, 14, 15^. Then, it is one of the important future problem to investigate the biological relevance of this phenomenon.

## Methods

We used the Izhikevich neuron model^9^ as a processing element of a neural network model, and constructed a neural network that is the same as the neural network used in the numerical experiment by Izhikevich^11^. Then, the neuron *i* in the neural network is expressed by Eq. (3):

3 $$\{\begin{array}{rcl}{\dot{v}}_{i}(t) & = & 0.04{v}_{i}{(t)}^{2}+5{v}_{i}(t)+140-{u}_{i}(t)+\sum _{j\in {S}_{i}}{w}_{ij}(t)H({v}_{j}(t-{\delta }_{ij})-30)\\ {\dot{u}}_{i}(t) & = & a(b{v}_{i}(t)-{u}_{i}(t)),\end{array}$$

When *v*_*i*_(*t*) ≥ 30 [mV], neuron *i* fires and the values of *v*_*i*_(*t*) and *u*_*i*_(*t*) are reset as follows:

$$\{\begin{array}{lll}{v}_{i}(t) & \leftarrow  & c,\\ {u}_{i}(t) & \leftarrow  & {u}_{i}(t)+d.\end{array}$$

where *t* is time, *v*_*i*_(*t*) is the membrane potential of the neuron *i*, *u*_*i*_(*t*) is the membrane recovery variable of neuron *i*, *S*_*i*_ is a set of neurons that are connected to the neuron *i*, *w*_*ij*_ is a synaptic weight from neuron *j* to neuron *i*, *δ*_*ij*_ is a conduction delay from neuron *j* to neuron *i*, *a*, *b*, *c*, and *d* are parameters, and *H*(*x*) is a step function (*H*(*x*) = 0 if *x* < 0, and *H*(*x*) = 1 if *x* ≥ 0). When *v*_*j*_ ≥ 30 [mV], that is, when neuron *j* connecting to neuron *i* fires, the value of the synaptic weight *w*_*ij*_ is applied to neuron *i*.

The neural network consists of 800 excitatory neurons and 200 inhibitory neurons. In this paper, we used regular spiking neurons (*a* = 0.02, *b* = 0.2, *c* = −65, *d* = 8) as excitatory neurons, and fast spiking neurons (*a* = 0.1, *b* = 0.2, *c* = −65, *d* = 2) as inhibitory neurons. Each neuron has 100 synapses connected to other neurons. Every excitatory neuron connects onto 100 neurons that are randomly chosen from among all neurons, while every inhibitory neuron connects onto 100 neurons that are randomly chosen from among excitatory neurons.

Conduction delays among neurons are random integers between 1 [ms] and 20 [ms]. The excitatory connection obeys the STDP learning rule at every one second. The maximum value of LTP, *A*_+_, is 0.1 and the maximum value of LTD, *A*_−_, is 0.12, because there are several experimental evidences that LTD of STDP window is larger than LTP^12, 13, 16^. About the value of *τ*, several studies by physiological experiments report that the time constant is estimated at near 10 [ms]^17–19^. In our study, we consider the effect of the domamine that reduces the value of *τ* ^8^. Although the amount of such doperminergic effects is quantitatively unknown, the value of *τ* needs to be smaller than 10 [ms] to reproduce the dopaminergic effects and we used those values. The initial values of the synaptic weights are set to 6, the maximum value is limited to 10, and the minimum value is limited to 0.

The excitatory connections are updated every second by Eq. (4):

4 $${w}_{ij}(t)={w}_{ij}(t-1)+\sum _{{t}_{i}=t-1}^{t}{\rm{\Delta }}{w}_{ij}({\rm{\Delta }}{t}_{ij}),$$

where Δ*w*_*ij*_(Δ*t*_*ij*_) is defined by Eq. (1) and depends on *t*_*i*_ and *t*_*j*_. This is because we followed the studies of Izhikevich^11^ and Morrison^20^. It is known that synaptic weights change due to 50–60 pairs of spikes^21, 22^, that is, synaptic weights change with a pace of seconds. Therefore we updated the connections once per second. Inhibitory connection weights are fixed to −5. A randomly chosen neuron receives a pulse of 20 [mA] every 1 [ms] as a random input. We used Euler’s method for numerical integration of Eq. (3) with the time step of the integration 0.5 [ms] by using C language. With these experimental conditions, we changed the value of the parameter *τ* to reproduce dopaminergic modulation, and investigated a time series of firing rates and synaptic weights. Source codes used in numerical experiments in this paper are available at ModelDB (http://senselab.med.yale.edu/ModelDB/showModel.cshtml?model=225818).

## Electronic supplementary material

Supplementary Information
