# Supplementary material for: Reproducing Infra-Slow Oscillations with Dopaminergic Modulation
Source: Sci Rep. 2017 May 25;7:2411. doi: 10.1038/s41598-017-02366-z (PMC5445087; doi:10.1038/s41598-017-02366-z)
Supplement: Supplementary file 1 — Supplementary Information [file 41598_2017_2366_MOESM1_ESM.pdf]

Supplementary Information for Reproducing Infra-Slow Oscillations with Dopaminergic Modulation

Toshihiro Kobayashi[†], Yutaka Shimada[†,‡], Kantaro Fujiwara[†,‡], Tohru Ikeguchi[†,‡]

† Department of Management Science, Graduate School of Engineering, Tokyo University of Science, Niijuku, Katsushika-ku, Tokyo, Japan
tkobayashi@hisenkei.net

‡ Department of Information and Computer Technology, Faculty of Engineering, Tokyo University of Science, 6-3-1 Niijuku, Katsushika-ku, Tokyo, Japan

Reproducing ISO by modulating the maximum values of LTP and LTD

In our study, we fixed the values of parameter A_+ and A_- ($A_+ = 0.1$, $A_- = 0.12$), and only changed the value of parameter τ in Eq. (1).

$$\Delta w_{ij}(\Delta t_{ij}) = \begin{cases} A_+ \exp(-\frac{\Delta t_{ij}}{\tau}) \ (\Delta t_{ij} > 0), \\ -A_- \exp(\frac{\Delta t_{ij}}{\tau}) \ (\Delta t_{ij} < 0), \end{cases} \tag{1}$$

where $\Delta t_{ij} = t_i - t_j - \delta_{ij}$, t_i is the firing time of the postsynaptic neuron i, t_j is the firing time of the presynaptic neuron j, δ_{ij} is the conduction delay from neuron j to neuron i, A_+ is the maximum value of LTP, A_- is the maximum value of LTD, and τ is the time constant of LTP and LTD.

The reason why we only changed the value of τ in numerical experiments is that we assume that the essence of generation mechanism of infra-slow oscillation is the width of the learning window in temporal direction. As shown in the main text (Fig. 1), we can explain the mechanism simply only by changing τ. However, it is also important to see whether ISO can be reproduced by STDP under physiologically plausible condition, then, we show results of STDP learning with large A_+ and A_-, and small τ.

According to Ref. [1], by activation of D1-type receptor, larger LTP and LTD are induced with a small timing difference, and smaller LTP and LTD are induced with a larger spike-timing difference. Namely STDP window is enlarged in vertical (change in synaptic weight) direction and narrowed in horizontal (temporal) direction. Therefore, we used larger A_+ and A_- ($A_+ = 0.2$, $A_- = 0.24$) and narrowed the STDP window by making τ small. Then, when $\tau = 2.2$ [ms], we reproduced ISO (Supplementary Fig.1).

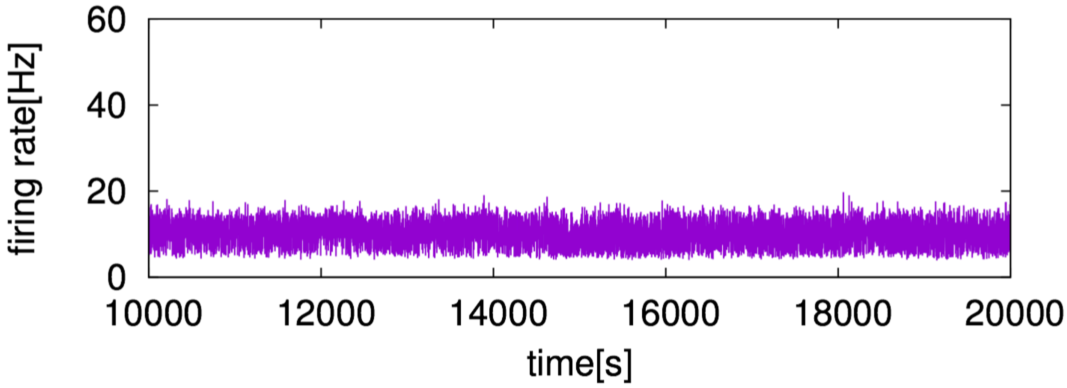

(a) $A_+ = 0.1, A_- = 0.12, \tau = 10$

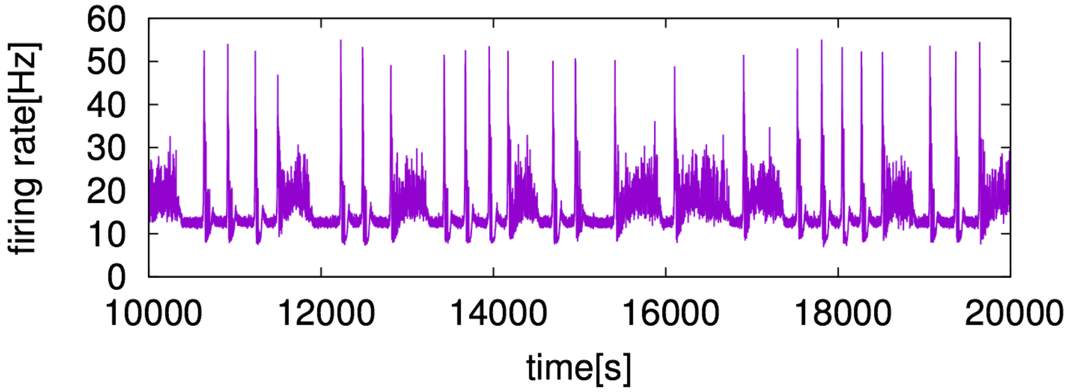

(b) $A_+ = 0.2, A_- = 0.24, \tau = 2.2$

Supplementary Figure 1: Temporal change in firing rates. The horizontal axis is time[s] and the vertical axis is firing rates. (a) The change without dopaminergic effect ($A_+ = 0.1, A_- = 0.12, \tau = 10$) and (b) the change with dopaminergic effect ($A_+ = 0.2, A_- = 0.24, \tau = 2.2$). ISO is reproduced with the parameter considering dopaminergic effect.

References

[1] Kechun Yang and John A. Dan, Dopamine D1 and D5 Receptors Modulate Spike Timing-Dependent Plasticity at Medial Perforant Path to Dentate Granule Cell Synapses, The Journal of Neuroscience, **26**, 34(48): 15888-15897, 2014.
